# Impingement in total hip arthroplasty: A geometric model

**DOI:** 10.1177/09544119211069472

**Published:** 2022-02-11

**Authors:** Gregory M Pryce, Bismaya Sabu, Mazen Al-Hajjar, Ruth K Wilcox, Jonathan Thompson, Graham H Isaac, Tim Board, Sophie Williams

**Affiliations:** 1 Institute of Medical and Biological Engineering, University of Leeds, Leeds, UK; 2 DePuy Synthes Joint Reconstruction, Leeds, UK; 3 Wrightington Wigan and Leigh NHS Trust, Wigan, UK

**Keywords:** Total hip arthroplasty, impingement, geometric model, surgical orientation, polyethylene

## Abstract

Total Hip Arthroplasty (THA) is one of the most common and successful surgical interventions. The survivorship at 10 years for the most commonly used systems is over 95%. However, the incidence of revision is usually much higher in the 0–1 year time period following the intervention. The most common reason for revision in this early time period is dislocation and subluxation, which may be defined as complete or permanent, and partial or temporary loss of contact between the bearing surfaces respectively. This study comprises the development of a geometric model of bone and an in situ total hip replacement, to predict the occurrence and location of bone and component impingement for a wide range of acetabular cup positions and for a series of frequently practiced activities of daily living. The model developed predicts that anterior-superior component impingement is associated with activities that result in posterior dislocation. The incidence may be reduced by increased cup anteversion and inclination. Posterior-inferior component impingement is associated with anterior dislocation activities. Its incidence may be reduced by decreased cup anteversion and inclination. A component impingement-free range was identified, running from when the cup was positioned with 45° inclination and 25° anteversion to 70° inclination and 15°–20° anteversion.

## Introduction

Total Hip Arthroplasty (THA) is one of the most common and successful surgical interventions. Estimating the annual total number of hip replacements worldwide is difficult. Kurtz et al.^1^ suggested this figure to be 959,000 primary and revision hip procedures. In 2019 in the UK (excluding Scotland) alone there were 101,384 primary and 8245 revision cases.^2^ The survivorship at 10 years for the most commonly used systems is over 95%. However, the incidence of revision is usually much higher in the 0–1 year time period following the intervention compared with the 1–10 year period.^2^ The most common reason for revision in this early time period is dislocation and subluxation, which may be defined as complete or permanent and partial or temporary loss of contact between the bearing surfaces respectively. Within the first year, this occurs in 2.50 cases per 1000 patients, much higher than other factors. The revision rate for mal-alignment occurs at a rate of 0.73 cases per 1000 patients, and it is likely that this is linked to subluxation and dislocation.^2^

The causes of dislocation and subluxation are multi-factorial and include poor positioning of components and/or variation of pelvic tilt^3–7^; inadequate soft tissue tension^8–12^; and low head-neck ratio limiting range of motion.^13–17^ All of these causes are at least in part driven by inadequate range of motion leading to impingement.

There are three possible types of impingement that can be investigated. There are component-component (or prosthetic), bone-bone and bone-implant impingement. Component-component impingement has been reported to be influenced by implant design^3,6,18–20^ and surgical positioning of the implant.^15,20–23^ Whereas, bone-bone impingement has been reported to be affected by variation in offset of the implant^24^ and variation in bone geometries.^25^ Whilst this is a collision of hard anatomical features, it may be compounded by changes in the relative position of the femur and pelvis following a hip replacement. Clinically interposition (or impingement) of the capsular and muscular tissue between prosthetic components and/or bone may also occur.

Finite element analysis (FEA) and geometric (or three-dimensional rigid body) modelling have been carried out to investigate both prosthetic and bone impingement but these both have limitations.^7,20,23,26–30^ Increased computational times have meant that the majority of the FEA studies that have investigated impingement typically have not included bone geometries.^7,26,28,30^ The majority of geometric models used to study impingement have included both implant and bone geometries,^20,27,29,31^ which has permitted the investigation of both component and bone impingement. A limitation of geometric models is that they are not able to consider the contact forces applied to the joint, and therefore the assessment of the severity of rim damage due to impingement is challenging. Geometric models are also not capable of considering any restraint provided by the supporting structures (such as muscles, tendons and ligaments). Consequently, the head and cup are typically constrained to be concentric to each other; therefore, any separation of the head due to microseparation^32^ or subluxation secondary to impingement^7,26,28,30,33^ is not able to be considered.

A major limitation of previous geometric models to assess impingement has been the manner in which joint motion was applied to the model, by moving the hip through one axis of motion and fixing the others.^20,23,27,34,35^ This methodology does not accurately simulate the joint motion of clinically relevant activities, because all three axes of motion of the hip vary when performing different activities of daily living.^36,37^ Models which use clinically relevant activities are more likely to predict both the incidence and location of impingement.

Acetabular cup orientation affects the incidence and location of impingement, previous computational studies have only considered a limited range of orientations.^7,23,26^ A clinical study by Lewinnek et al,^38^ proposed a range of acetabular cup orientations (30°–50° inclination and 5°–25° anteversion) which was driven by the incidence of dislocation and did not consider the incidence of subluxation caused by impingement. Although the study by Lewinnek was performed over 40 years ago and devices have changed since the study was performed (such as increased use of larger femoral head sizes), their recommended safe zone is still considered in clinical practice today.

Previous computational studies have reported on the occurrence of component impingement; however, the location of any consequential damage has not been widely reported. Therefore, the predictions from these studies cannot be correlated to damage reported in retrieval studies.

The aim of this study was to develop a geometric (or rigid body) model of bone and an in situ total hip replacement and apply a set of activities of daily living, to determine if the occurrence and location of bone and component impingement could be predicted for a clinically relevant range of acetabular cup positions.

## Materials and methods

A geometric model of a hemi-pelvis and superior femur was created using a computer-aided design (CAD) modelling software package (SOLIDWORKS 2016, Massachusetts, USA), and a THA was virtually implanted into the bone to achieve restoration of joint centre. A series of motions associated with ‘dislocation prone’ activities were applied to the model.^36^ The incidence and location of component and bone impingement was assessed for a clinically relevant range of acetabular cup positions.

### Development of the geometric model

**Figure 1.**
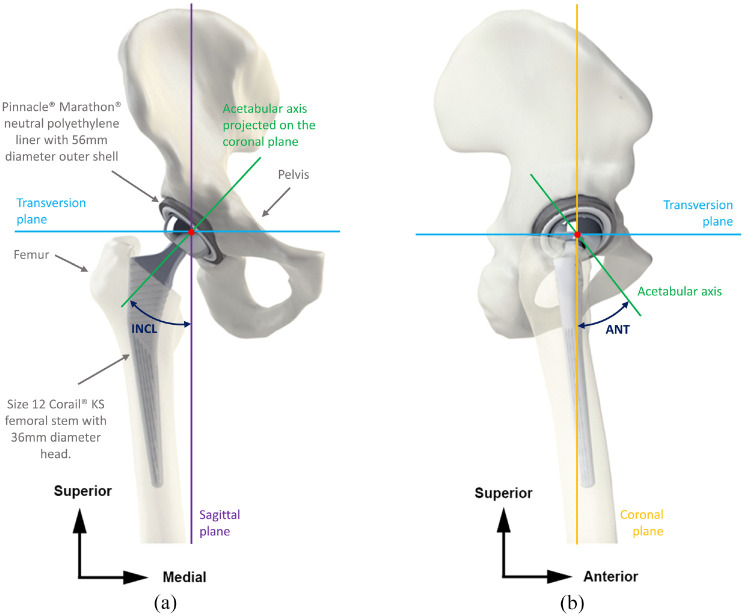
Geometric model produced from bone surface geometries with a THA components virtually implanted, with key components labelled (ANT = cup anteversion; INCL = cup inclination): (a) coronal view and (b) sagittal view.

Three-dimensional models of the right hip joint (right hemi-pelvis and right femur) bone surface geometries (generated from computed tomography scans) (Figure 1), were obtained from the BEL repository.^39^ The hip was from a 38 year old male whose bone was described as healthy at the time of death. The geometries of femoral and acetabular components implanted were based on a widely used THA system. This comprised a size 12 Corail^®^ KS (uncollared) cementless stem, 36 mm diameter Articul/eze Ultamet^®^ femoral head, Marathon^®^ (cross-linked polyethylene) neutral acetabular liner and a 56 mm diameter Pinnacle^®^ acetabular shell (Figure 1). All components were manufactured by DePuy Synthes (Leeds, UK). The models were developed from CAD files and engineering drawings provided by the manufacturer. For ease of modelling some features not associated with the impingement scenario being investigated were simplified, for example removing the locking mechanism features from the liner and shell. Creep or wear of the liner was beyond the scope of this model which considers only short-term effects.

To orientate the hemi-pelvis and femur in a neutral (or stance) position, anatomical planes, and the centre of rotation (COR) of the pelvis and femur were determined. The COR of the acetabulum and femoral head were defined by fitting a sphere 56 mm in diameter in the acetabulum and 50 mm in diameter to the natural femoral head. Anatomical planes of the pelvis were determined by defining the anterior pelvis plane (APP), as described by Kubiak–Langer et al.^40^

The natural femoral head was removed at the base of the neck, and the femoral stem was positioned to conform with the geometry of the native femur, which resulted in the stem being orientated at 15° anteversion, 6° adduction and 6° flexion. The centre of the prosthetic head was aligned to the centre of native head. Material was removed from the native acetabulum to simulate the process of reaming the acetabulum. A sphere with the same diameter of the outer shell was used to remove the material; therefore, the acetabular cup fitted tightly within the reamed acetabulum. The centre of the sphere was translated, so that the outer surface of the cup was in contact with the true floor of the acetabulum. This resulted in the COR of the cup moving in a medial-superior direction 6 mm from the COR of the natural acetabulum. Component positioning was confirmed as clinically relevant by a consultant orthopaedic surgeon (TB).

**Figure 2.**
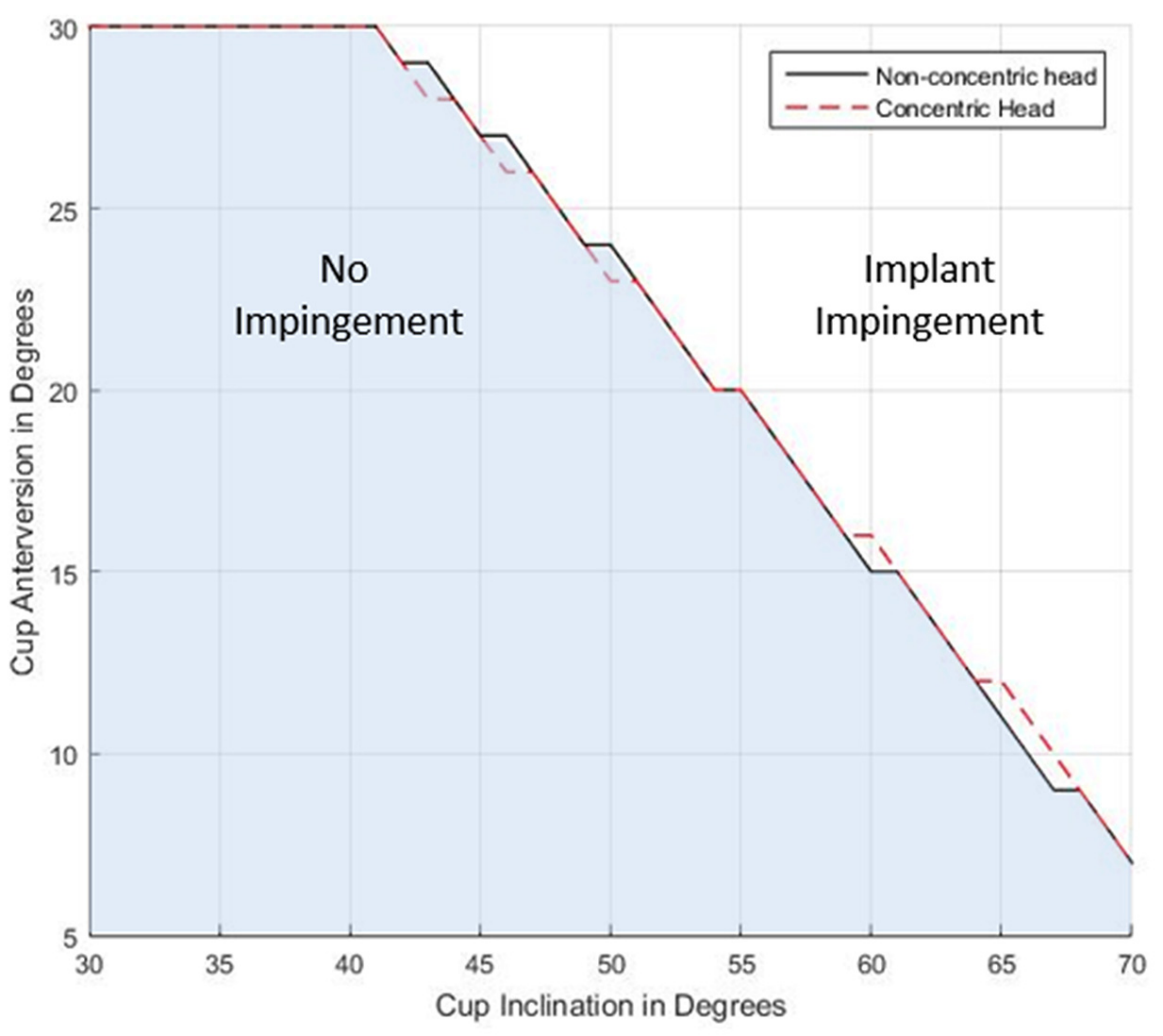
Occurrence of component-component impingement when the femoral head is in contact with the liner (nonconcentric) (solid black line) and femoral head is concentric to the liner (dashed red line), across a range of acetabular cup orientation (inclination and anteversion) when performing the STOOP activity.

A sensitivity analysis was completed on the model to investigate the effect of constraining the CORs of the head and cup, so they were coincident (concentric model) compared with the more clinically relevant situation of the head maintaining contact with the liner at impingement (non-concentric model). Contact between the head and liner was achieved by translating the COR of the head in a medial-superior direction to the point just before contact. The analysis was performed by applying the activity of bending over at the waist to reach for an object on the floor while standing (STOOP) to the model. The results from analysis showed that the two conditions resulted in very similar predictions of impingement, with only a few cup orientations resulting in different predictions of impingement (Figure 2). Hence for simplicity, in this model the COR femoral head and acetabular cup were co-incident (concentric model).

To orientate the femur in a neutral position relative to the pelvis, the anatomical planes of the femur were made parallel with the anatomical plane of the pelvis, and this defined the initial position of the femur in this study.

### Model inputs (motions – activities of daily living)

**Figure 3.**
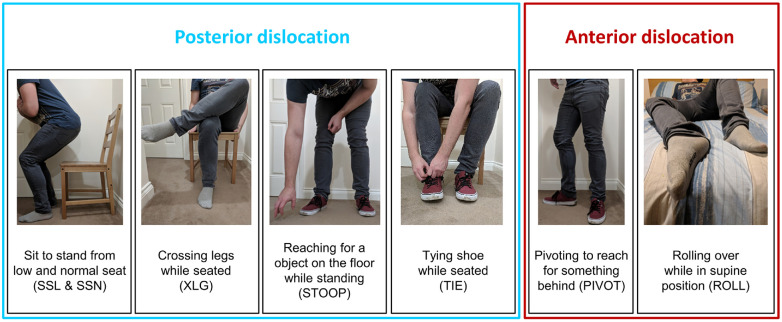
Images demonstrating the different activities of daily living from Nadzadi et al.^36^

Joint motions applied to the model were taken from kinematic data of activities of daily living that are associated with dislocation of a THA, as described by Nadzadi et al.^36^ and shown in Figure 3. Five activities were associated with posterior dislocation and two with anterior dislocation. The posterior dislocation-prone activities were: sit-to-stand from a low seat (SSL), sit-to-stand from a normal height chair (SSN), leg crossing while seated (XLG), shoe tying while seated (TIE), bending over at the waist to reach for an object on the floor while standing (STOOP). The anterior dislocation-prone activities were: rotating the upper body away while standing (PIVOT) and rolling over while in the supine position (ROLL). This data was defined as the position of the femur relative to the pelvis, therefore included the motions of the pelvis when performing the activities. The joint motions of the activities were the data of a single subject that was representative of the performance of five male and five female healthy subjects with an average age of 49.7 years (range 44–59) and mean weight of 77.3 kg (range 40.0–122.7).^36^

For reference, motions occurring during a standard walking cycle as defined in Hip98 data, which comprised four patients with an average weight of 84 kg,^37^ were applied to the same bone geometries used in the rest of the study.

Joint motion was defined in all three axes of rotation of the hip; flexion/extension, adduction/abduction and internal/external rotation, defined as positive/negative degrees respectively. Motion was applied in the model by altering the position of the femur about the COR while the pelvis remained fixed. The position of the femur was described by the location of two reference points (distally and laterally from the head), and these locations were defined as three-dimensional (3-D) coordinates. These coordinates were determined by converting the kinematic data using a series of rotation matrices for each of the activities.

### Model inputs (variation of acetabular cup position)

To investigate the effect of surgical variation, the positioning of the acetabular cup relative to the pelvis, the radiographic inclination and anteversion of the cup was varied.^41^ Ranges including the extremes of clinical practice were considered, which comprised inclinations of 30°–70° and anteversions of 0°–50° in increments of 5°.

### Model outputs

Impingement was identified in the model through the use of the ‘interference detection’ function in the CAD software package. This function determined when interference (or overlap) occurred in the model, and this was able to detect low volumes (>1 mm^3^) of interference of the geometries. This was used to assess the range of motion up to impingement when performing an activity of daily living. At the point of impingement, the joint position (i.e. flexion/extension angle), the type of impingement (component or bone) and location of the component impingement on the liner rim was recorded.

### Verification

A verification process was undertaken on the developed model, to determine whether inputs applied to the model (joint motions of the activities and cup orientations) and the output data generated from the model were accurate.

Verification of both the joint motions and cup orientations were performed by applying a set joint position and acetabular cup orientation to the model. The ‘measurement tool’ feature in the CAD software package was used to measure the angles between the reference planes and axes that are associated with the axes of rotations and cup orientations. To assess if there was any deviation between the applied inputs and the measured values, the geometric model was visually inspected to ascertain the occurrence and location of impingement. The verification of the inputs (joint motion and cup orientation) found that the maximum deviation was less than ±0.01° and this was deemed to be acceptable for this study.

## Results

The data associated with this paper are openly available from the University of Leeds data repository.^42^

### Impingement predictions

**Figure 4.**
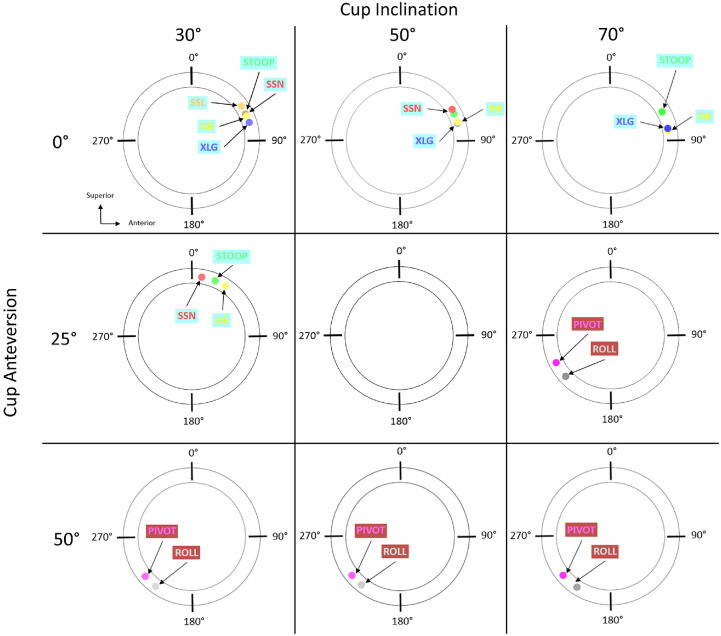
Plan views of acetabular cups at different cup inclination (30°, 50°, 70°) and anteversion (0°, 25° and 50°). The location of impingement predicted by the geometrical model is marked for the different activities XLG, SSN, SSL, TIE, STOOP, ROLL and PIVOT). Activities associated with posterior dislocation are highlighted in cyan. Those associated with anterior dislocation are highlighted in maroon. Small degree marker represents angular position clockwise from superior.

**Figure 5.**
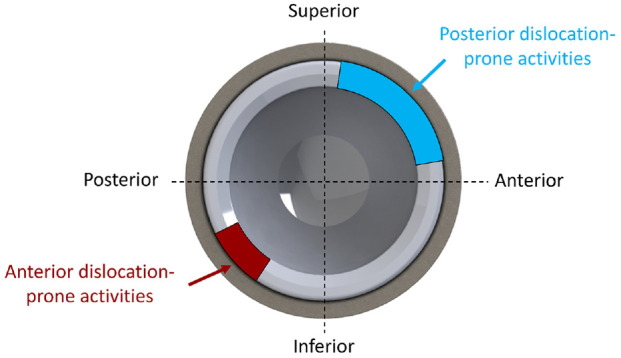
Schematic of the acetabular cup, demonstrating the location of impingement relative to the liner rim, across all of activities of daily living investigated in this study. Cyan corresponds to location of anterior impingement and maroon corresponds to the location of posterior impingement.

The seven activities described in section 2.3 and illustrated in Figure 3 were applied, and the location of component-component impingement was identified for various cup orientations as shown in Figure 4. Anterior impingement occurred during activities prone to posterior dislocation (STOOP, TIE, XLG, SSL, SSN). Impingement took place between the rim of the cup and the neck of the stem in the superior-anterior quadrant of the liner (0°–90° around the circumference of the cup rim) (Figure 5). All five activities produced impingement at 30° inclination and 0° anteversion. Increasing inclination and anteversion both tended to reduce the occurrence of anterior component impingement. Posterior component-component impingement was associated with activities prone to anterior dislocation (PIVOT and ROLL). It was located in the posterior-inferior quadrant of the liner (180°–270° around the circumference of the cup rim) (Figure 5). Decreasing anteversion and to a lesser extent inclination, tended to reduce the incidence of posterior component-component impingement.

Bone-bone impingement occurred between the anterior osseous femoral neck and anterior-inferior iliac spine for anterior impingement (or posterior dislocation-prone) activities (STOOP), and between the posterior osseous femoral neck and ischium for posterior impingement (or anterior dislocation-prone) activities (ROLL). Component-bone impingement did not occur in this study.

**Figure 6.**
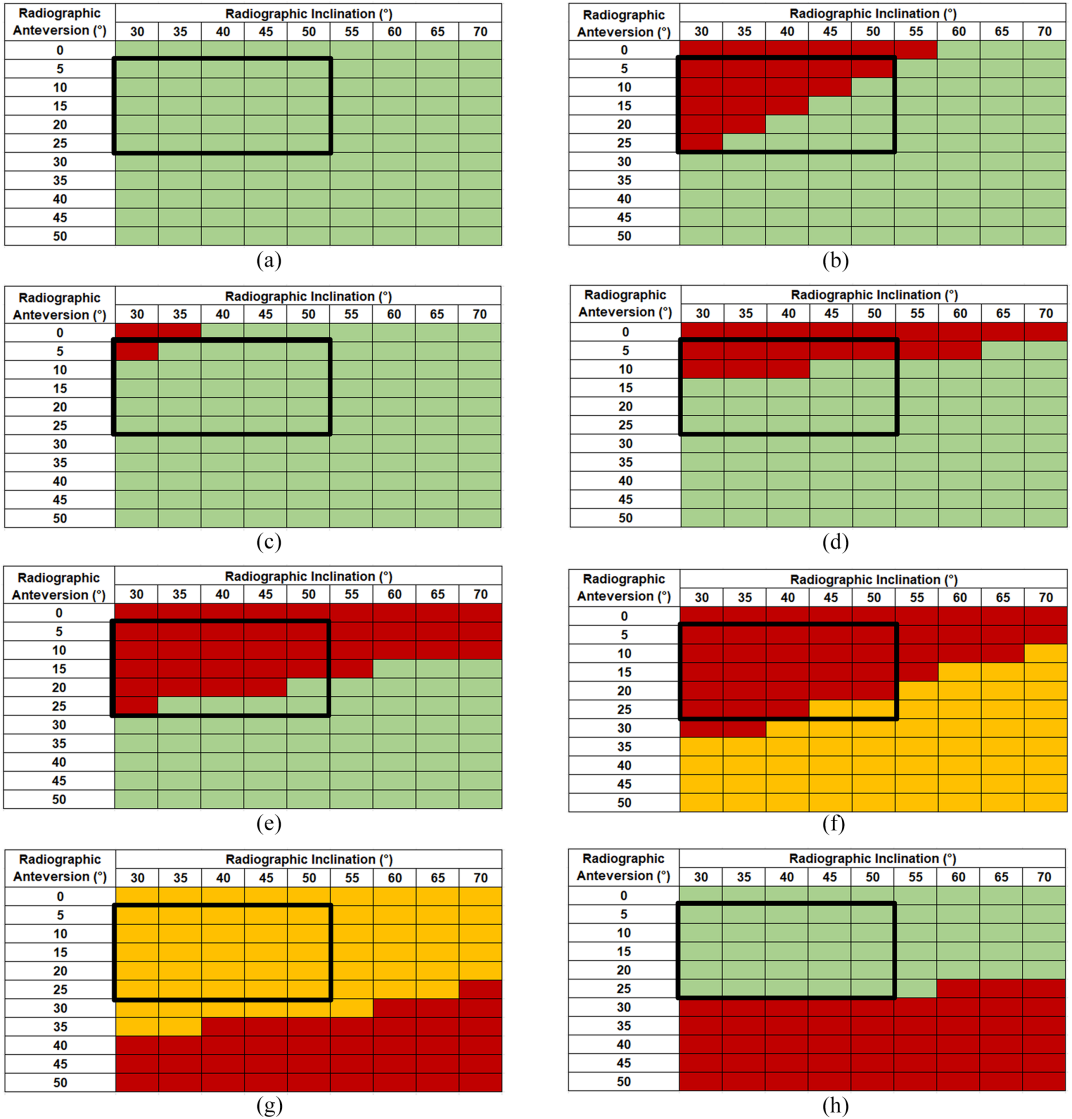
Occurrence of component-component (red) and bone-bone (amber) impingement in THA for a range of inclination and anteversion angles under different activities. (a) walking at normal speed, (b) SSN, (c) SSL, (d) XLG, (e) TIE, (f) STOOP, (g) ROLL and (h) PIVOT. Green indicated no impingement. The cup orientations within the box delineated with bold black lined box define the ‘safe zone’, as described by Lewinnek et al.^37^

The incidence of component-component and bone-bone impingement (whichever occurred first) predicted by the model, was recorded as a matrix of cup anteversion and inclination, as shown in Figure 6. For reference the range historically defined by Lewinnek et al.^38^ as the ‘safe zone’ has been superimposed onto the matrix.

When a standard walking cycle (Hip98 data) was applied, the model predicted that impingement would not occur in any of the positions assessed (Figure 6(a)).

The ‘sit-to-stand from a low seat’ (SSL) and ‘sit-to-stand from a normal seat’ (SSN) activities (Figure 6(b) and (c)) were both predicted to cause anterior component impingement at low inclination and anteversion angles. However, somewhat counterintuitively, impingement was recorded for an increased range of cup orientations with the SSN activity compared to SSL.

‘Leg crossing’ activity (XLG, Figure 6(d)) resulted in anterior component impingement when the cup was positioned between 0° and 10° anteversion at 30° inclination, but only at 0° anteversion at 70° inclination. In contrast, ‘tying shoelaces’ (TIE, Figure 6(e)) was predicted to cause anterior impingement across a larger range of anteversion (0°–25° at 30° inclination) but reducing at higher inclinations (0°–10° at 70° inclination).

When the ‘stooping to pick an object from the floor’ (STOOP) activity was modelled, either component or bone anterior impingement was observed across all of the cup orientations (Figure 6(f)). Component impingement occurred when the cup was orientated between 0° and 30° anteversion at 30° inclination. The range of anteversion at which impingement occurred reduced to 0° to 5° at 70° inclination. The opposite was true with bone impingement, the range of anteversion at which impingement occurred increasing from 35° to 50° at 30° inclination to 10°–50° at 70° inclination.

‘Roll in supine position’ (ROLL) activity resulted in either component or bone posterior impingement at all cup orientations considered (Figure 6(g)). Component posterior impingement was confined to relatively high anteversions (40°–50°) at low inclination (30°), but the range increased to 25°–50° at 70° inclination. Conversely bone posterior impingement occurred at 0°–35° anteversion at 30° inclination, reducing to 0°–25° at 70° inclination. ‘Pivoting for an object from behind’ (PIVOT) activity (Figure 6(h)), resulted in only component posterior impingement and was predicted to occur at higher anteversion angles 30°–50° at 30° inclination, but the range increased to 25°–50° at 70° inclination.

**Figure 7.**
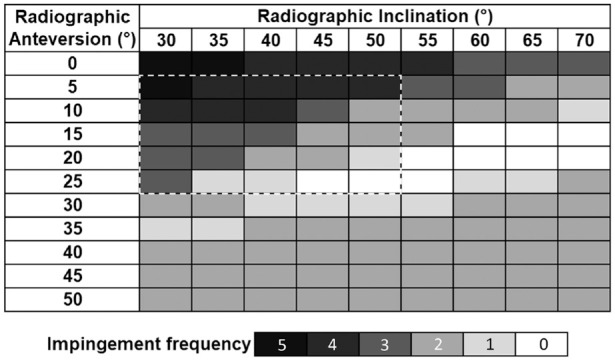
Frequency plot of the occurrence of only component-component impingement from a geometric model of a THA where the femoral stem was fixed and the acetabular cup inclination and anteversion were varied. Black represents the highest frequency of component impingement, and white represents the least frequency of component impingement across the seven of activities that were investigated. Cup orientations within the bold white dash-lined box is the ‘safe zone’ as described by Lewinnek et al.^37^

To provide an overall assessment of the likelihood of component impingement following application of the activities described for cup positions (Figure 6) a ‘frequency of impingement’ plot was developed (Figure 7). This represents the number of the seven activities which were predicted to result in only component impingement for a specific inclination and anteversion. A component impingement-free range was identified, running from when the cup was positioned with 45° inclination and 25° cup anteversion to 70° inclination and 15°–20° anteversion. Hence as cup inclination increased, a lower anteversion angle was required to prevent impingement. Relaxing the criteria to one activity resulting in impingement gives a range of orientations from 30° inclination and 35° anteversion to 70° inclination and 10°–20° anteversion.

## Discussion

This study used a geometric model to predict the incidence and location of both component-component and bone-bone impingement for a number of activities of daily living over a range of component orientations. The disadvantage is that unlike in Finite Element (FE) models, contact stresses and consequences of impingement (dislocation or subluxation of the joint) on the performance of the implant cannot be predicted. However, the use of this simpler model, which takes less than a minute to run, is advantageous because it permits a wide range of variables to be investigated including different implant designs and bone geometries. Joint motion from hip kinematic data generated in a gait study was applied, allowing inclusion of a range of activities. Using kinematic data additionally meant that all three axes of motion of the hip were considered simultaneously; therefore, simulating joint motions of activities of daily living. Most geometric models used to assess impingement applied motion by moving the model through one axis of joint motion whilst fixing the other two axes.^20,34,35,43^

This study has several limitations. The activities were applied only to one hip geometry, one design of implant and the motion data used was the mean of 10 individuals and gives no indication of the variability across a population; therefore, the predictions from the model should be used with caution to influence clinical practice. Furthermore, whilst dislocation is the most common reason for revision in the 0–1 year time period, in the longer term other factors such as wear and loosening become more common. Placing components solely to reduce impingement may lead to problems later in the component life-cycle. The centre of the head was required to be constrained relative to the pelvis; therefore, the model was not able to consider any separation of the head, as result of microseparation^32^ or subluxation secondary to impingement.^7,26,28,30,33^

Furthermore, the current study did not consider the influence that the orientation of the pelvis has on the surgical positioning of the cup, which could also affect the occurrence of impingement. It has been reported that there is a relationship between the orientation of the pelvis and the surgical positioning of the cup, as variation in sagittal pelvic tilt influences the functional anteversion of the acetabular cup.^4,5,44^ Pelvic tilt varies between supine, standing and sitting, and the range of tilt has been reported to be influenced by the stiffness of the lumber spine.^45,46^ Consequently, the mobility of the pelvis should also be considered when selecting the optimal surgical positioning of the cup; therefore, future studies should consider this mechanism.

In common with other models investigating impingement of THAs, this model did not consider the soft (or capsular and muscular) tissues structures that surround the hip joint. These structures reduce the free space between the implant and/or bone; therefore, it would be expected to reduce the maximum range of motion and impingement could occur before predicted by the model. Conversely other structures may provide joint stability thereby reducing the adverse effects of impingement. Whilst including soft tissues in the model would improve the clinical relevance of the predictions, modelling of soft tissues is complex, and was beyond the scope of the study. Variations in the positioning of the stem, implant design and size, bone geometry (i.e. different patient anatomies), and the position of the cup within the acetabulum (medialisation) were not investigated in this study. Varying these factors may have significant impact on risk of impingement. In this study the analysis has focused on component impingement primarily because the anatomy used was from one patient, and it would be inappropriate to place too much emphasis on bone impingement results.

At a simplistic level, the model predicts generally what would be expected. Specifically, activities that result in posterior dislocation are associated with anterior impingement. Increasing anteversion and to a lesser extent increasing inclination reduced the incidence of component impingement. The converse is true for anterior dislocation although the effects of inclination are less marked. Bone impingement was confined to the activities of ROLL and STOOP. In both of these activities the model predicted that with this data set, impingement (either bone or component) took place at all orientations of component placement. These activities represent the extremes of range of motion and compared to normal walking are carried out relatively infrequently.

Elkins et al.^33^ reported that bone impingement occurred when performing a deep squat after 105° of flexion, and was between the anterior osseous femoral neck and anterior-inferior iliac spine. This was also observed in this study following a high flexion activity (STOOP). In a high external rotation activity (ROLL) impingement was between the posterior osseous femoral neck and ischium of the pelvis, which is in agreement with Shoji et al.^25,47^ Shoji et al.^47^ also reported bone impingement to be affected by variation in bone morphologies of patients, as larger bone morphologies are likely to increase the risk of bone impingement.

An impingement-free range of cup orientations are highlighted in Figure 7 (where none of the activities of daily living used induced component impingement). As previously discussed Lewinnek et al.^38^ proposed a ‘safe zone’ and this has been superimposed on Figure 7. This shows that for component impingement, only acetabular cups placed at 45-50° inclination and 25° anteversion would lie both within this ‘safe zone’ and have no impingement when undertaking these activities. However, this ‘safe zone’ was attributed to the clinical incidence of dislocation, and impingement does not necessarily lead to dislocation. Studies by McCollum and Gray^48^ and Barrack et al.^49^ considered the influence that acetabular cup orientation has on the occurrence of dislocation and impingement, both reported a slightly increased overlap with the impingement-free range predicted by this study. Clinical ranges of cup orientations have been reported by Danoff et al.^50^ to be 18°–80° inclination and −16° to 48° anteversion, and a study by Rittmeister and Callitsis^51^ reported range of 23°–67° inclination and 1°–34° anteversion. Both studies observed ranges greater than the recommended ‘safe zone’.^38^ This demonstrates that the range of inclination and anteversion chosen in this study (30°–70° and 0°–50°, respectively) were not excessive.

In agreement with the current study, the risk of component impingement has been suggested to be decreased by a high cup inclination angle.^15,23^ However, excessive inclination (or abduction) of the acetabular component (>50°) has been reported to increase the risk of edge loading, causing undesirable tribological and mechanical outcomes.^52–56^ Such studies have led to a reduction in target inclination angles, which from this study would pre-dispose a joint construct to impingement, when compared to higher inclination angles (i.e. greater than 45°). These contradictory results emphasise the need to consider a number of factors when optimising component placement.

Clinically posterior dislocation has been reported to occur more frequently than anterior dislocation following THA.^57–59^ This factor should also be considered when selecting the optimal surgical positioning and design of implant used clinically.

Further investigations are proposed and will include a broader range of input data sets (e.g. bone geometries, activities and implant designs), to determine if the predictions generated by the model are more generally true.

## Conclusion

This study demonstrates the principle of the model and shows that it can be used to rapidly assess a range of variables.

Under the input conditions used in this study, the geometric model developed predicted that anterior-superior component impingement is associated with activities that result in posterior dislocation. The incidence may be reduced by increased cup anteversion and to a lesser extent increased inclination. Posterior-inferior component impingement is associated with anterior dislocation activities. Its incidence may be reduced by decreased anteversion and to a lesser extent by reduced inclination. A component impingement-free range was identified running from when the cup was positioned with 45° inclination and 25° cup anteversion to 70° inclination and 15°–20° anteversion.

Whilst in this study the model used specific activities of daily living, a single bone geometry and a single implant design, different input parameters could be applied in the future to evaluate a wider range of clinical scenarios.
